# Depression, Anxiety and Stress Among Students at the University of Pristina-Kosovska Mitrovica, Kosovo and Metohija, Serbia

**DOI:** 10.3390/healthcare14070958

**Published:** 2026-04-06

**Authors:** Danijela Ilic, Jovana Milosevic, Jovana Todorovic, Zorica Terzic-Supic, Ilija Dragojevic, Mirjana Stojanovic-Tasic, Emilija Novakovic, Tijana Spasojevic, Svetozar Memarovic, Milivoje Galjak, Kristina Rakic, Mirijana Virijevic, Kristina Stevanovic, Jelena Stefanovic, Biljana Trajkovic, Andrija Milovic, Momcilo Mirkovic

**Affiliations:** 1 Faculty of Medicine, University of Pristina-Kosovska Mitrovica, 38220 Kosovska Mitrovica, Serbia; 2 Institute of Social Medicine, Faculty of Medicine, University of Belgrade, 11000 Belgrade, Serbia; 3 Institute of Mental Health, 11000 Belgrade, Serbia; 4 Clinic for Otorinolaryngology and Maxillofacial Surgery, University Clinical Centre of Serbia, 11000 Belgrade, Serbia; 5 Faculty of Medical Sciences, University of Kragujevac, 34000 Kragujevac, Serbia; 6 The College of Health Sciences, Academy for Applied Studies Belgrade, 11000 Belgrade, Serbia; 7 Clinic for Ophthalmology, Clinical-Hospital Centre ‘Zvezdara’, 11000 Belgrade, Serbia; 8 Clinical Hospital Centre Kosovska Mitrovica, 38220 Kosovska Mitrovica, Serbia

**Keywords:** mental health, students, depression, anxiety, stress

## Abstract

**Introduction:** The aim of this study was to examine the prevalence of scores indicating depression, anxiety and stress (<95th percentile of the score on each of the domains) among students at the University of Pristina-Kosovska Mitrovica and social and lifestyle characteristics associated with scores indicative of depression, anxiety and stress in this population studying in a post-conflict area. **Methods:** The cross-sectional study applying the non-probabilistic convenience sampling that included a total of 656 students of nine faculties who were present in the classes during the day of this study at the University of Pristina-Kosovska Mitrovica was conducted during the 2024/2025 school year. **Results:** A total of 9.3% had a score on the DASS-D scale, indicating severe or extremely severe depression, 19.6% had a score indicating severe or extremely severe anxiety, and 13.9% had a score indicative of severe or extremely severe stress. Our study showed the association of scores indicating depression with living in rural areas, average self-rated health, use of anti-anxiety medications, and mobile phone addiction. Our study showed the association of scores indicating anxiety and average self-rated health, use of anti-anxiety medications, score on social support scale, and score on state impulsivity scale. Our study showed the association of scores indicating stress with female sex, age in years, poor self-rated financial status, average self-rated health, use of anti-anxiety medications, and score on the state impulsivity scale. **Conclusions:** This study has shown a significant burden of psychological distress among students at the University of Pristina-Kosovska Mitrovica.

## 1. Introduction

Mental health is defined by the World Health Organization (WHO) as a ‘state of mental well-being’ that enables people to cope with stresses of life, realize their abilities, learn well and work well and contribute to their community’ [1]. Mental health is influenced by the different social and individual characteristics, which may change throughout one’s lifetime [1,2,3]. Individual factors significantly influencing mental health include personality traits, emotional skills, and consumption of alcohol, cannabis and other psychoactive substances, along with genetics and family history. Additional factors that certainly contribute to mental health and to the development of mental health issues are economic, political and environmental factors, such as financial constraints and living in poverty, exposure to violence, and inequality [1,4,5,6,7,8,9,10,11,12,13].

Depressive disorders are relatively common mental health disorders among adults in the general population, with an estimated prevalence of 4–7%, higher among females [14]. According to the Global Burden of Disease Study, in 2021, depressive disorders were the second highest cause of Years Lived with Disability (YLDs), associated with 56.3 million YLDs, with an increase of 36.5% from 2010 [15]. Depressive disorders are also one of the most common mental health issues among students [16]. Depressive disorders in this population can be associated with decreases in academic performance and dedication to studying, risks of not graduating, lifestyle issues like smoking, risky sexual behavior, alcohol and drug use, and worryingly increased risk of suicide [16]. Symptoms of depression among youth are shown to be more prevalent and more severe among females [17,18]. Additionally, for young adults, self-rated health may serve as a valid indicator of mental health status, as high depressive symptoms were associated with poor self-rated health among university students [19]. Financial hardships have previously been shown to be associated with symptoms of depression and anxiety [20], although a review on poverty and mental health showed no clear association between income and mental disorders [21]. Impulsivity has been found to be co-morbid with a variety of psychiatric disorders, especially with depression, in studies that examined both depression and impulsivity using the self-reported instruments [22].

Excessive mobile phone use, and specifically mobile phone addiction, and problematic internet use were found to be associated with depressive symptoms among youth [23,24,25].

Anxiety is also a mental health issue with a high burden worldwide, and the Global Burden of Disease Study from 2021 showed that anxiety is associated with 42.5 million YLDs [15]. Among students, it was shown that anxiety is the most common cause of need for mental healthcare services and is associated with diminishing academic performance [26]. The prevalence of anxiety among university students in different studies varies from 31% to 55% [27,28].

Generally, students are also at increased risk of stress, as they are facing constant life changes and instability. This period is marked by the transition from living with family and parents to living alone, along with educational and academic pressures and maintaining romantic partnerships [29]. Maintenance of social support can serve as a protective factor for this population, as a negative association between social support and depressive symptoms has been repeatedly shown [21,30,31,32,33]. Poor adaptive strategies to these stresses may be associated with the mental health strain and further lead to the development of mental health disorders such as depression and anxiety [29]. Common lifestyle issues, such as screen time, low physical activity and poor diet, are also associated with poor mental health outcomes among university students [34]. Conversely, compliance with healthy lifestyles, including regular physical activity, non-smoking, and non-risky alcohol consumption, is associated with overall mental well-being [35].

Kosovo and Metohija is a disputed region in southern Serbia that declared independence in 2008, but the independence is not recognized by Serbia and by the majority of the members of the United Nations (UN) [36]. This has caused the current status of the region to be stable; however, it is still unresolved [36]. The independence of the region followed the conflict that was most strongly pronounced during the late 1990s, and students currently studying in Kosovska Mitrovica may have been exposed to mass violence and displacements or are facing struggles and uncertainty in their daily lives, living in a politically charged environment, which all have been associated with mental health problems, including depression, anxiety and stress [37]. Life in conflict areas is associated with higher rates of a wide range of mental disorders, including an almost three times higher likelihood for the development of anxiety, depression, substance abuse, and post-traumatic stress disorder [38,39]. Among young adults, these symptoms may be accompanied by obsessive thoughts about their homes, self-imposed social distancing and somatic symptoms [39]. Moreover, life in post-conflict areas is associated with poverty, unemployment, violence, and overall unstable living conditions, all associated with lower quality of life and likelihood of the development of various mental health issues [40]. The University of Pristina-Kosovska Mitrovica is the only university in Kosovo and Metohija that functions as a part of the educational system in Serbia [41].

The prevalence of any mental health disorders in the general population of Serbia was 15.2%, while the prevalence of self-reported depressive symptoms among adults in Serbia was 4.3% in the Serbian National Health Survey in 2019. The prevalence among university and high school students was higher, reaching more than 30% for both depressive and anxiety symptoms [42,43]. In the 21st century, there has only been one study that examined mental health among Serbians in Kosovo, conducted on a representative sample of adults in four municipalities [44]. This study was conducted in 2009, with the mean age of the population of 44 years, and to the best of our knowledge, no study examined the mental health of young adults in this vulnerable area.

The aim of this study was to examine the prevalence of depression, anxiety and stress among students at the University of Pristina-Kosovska Mitrovica and social and lifestyle characteristics associated with depression, anxiety and stress in this population.

## 2. Materials and Methods

The cross-sectional study that included a total of 656 students at the University of Pristina-Kosovska Mitrovica from nine different faculties (faculty of physical education, natural and mathematical sciences faculty, faculty of technical sciences, teachers faculty, faculty of agriculture, faculty of economics, faculty of medicine, faculty of law, and faculty of philosophy) was conducted during the 2024/2025 school year. All students present at the faculty when the researchers conducted this study were invited to participate, applying convenience sampling in this study. In the absence of reliable prior data required for formal sample size calculation, to obtain an adequate sample size, we used Slovin’s formula (n = N/(1 + N × 10^3^) with a margin of error of 4% and N = 9000, which gave us a sample size of 585 students. All students were given the information about this study, its processes and aims and gave informed consent for participation in this study. The questionnaires were anonymous, and the data were analyzed only as the aggregate data. This study was approved by the ethical committee of the faculty of medicine, University of Pristina-Kosovska Mitrovica, where the lead author is based (No. 09-320212), and all methods were performed in accordance with the Declaration of Helsinki.

The study instrument was a questionnaire that consisted of the following sections: socio-demographic and socio-economic characteristics, lifestyle characteristics, mobile phone addiction, study engagement, physical activity, perceived social support, problematic internet use, sleep quality, state impulsivity, and emotional states of depression, anxiety and stress.

Section on socio-demographic and socio-economic characteristics referred to faculty at which the participants study, sex (male/female), age (in years), type of residence (urban/rural), height (in centimeters), weight (in kilograms), grade point average, relationship status (in a relationship or married/single), self-rated financial status (very poor, poor, average, good, very good), familial relationships (very poor, poor, average, good, very good), and self-rated health (very poor, poor, average, good, very good). For this study, in the variables self-rated financial status, familial relationships and self-rated health, the categories ‘very poor’ and ‘poor’ were merged into ‘poor’, while the categories ‘very good and good’ were merged into ‘good’. Body mass index (BMI) was calculated based on the self-reported height and weight as the ratio between the weight in kilograms and the square of height in meters (kg/m^2^) [45,46]. The section on lifestyle characteristics referred to tobacco smoking (yes/no), use of electronic cigarettes (yes/no), use of any tobacco-heating products (yes/no), cannabis use (yes/no), alcohol consumption (yes in the past year/yes in the past month/not at all), binge drinking (yes in the past year/yes in the past month/not at all), use of any anti-anxiety medication (yes/no), time spent on social media per day in hours, and time spent playing video games per day in hours. Binge drinking was defined as having more than 60g of pure ethanol (1500 mL of beer, 700 mL of wine or 150 mL of hard liquor) on at least one occasion. Alcohol consumption was defined as any alcohol consumption in the past year, while binge drinking was examined as binge drinking in the past month; the students who reported binge drinking in the past year and not in the past month were classified as non-binge drinkers, in line with the most common definition of binge drinking [45,46,47].

Mobile phone addiction was examined using the smartphone addiction scale—short version, a ten-item scale referring to disruption in daily life, withdrawal symptoms, relationships centered online, excessive use and tolerance, with answers ranging from 1—strongly disagree to 6—strongly agree [48]. The score on the scale varies from 10 to 60, and higher scores correspond with higher levels of mobile phone addiction. The scale has different cut-offs for male and female participants. The cut-off for mobile phone addiction for male participants is 31, and for female participants is 33 [48]. Cronbach’s alpha for the smartphone addiction scale—short version in our sample was α = 0.861.

Study engagement was examined using the study engagement scale, which is a nine-item instrument that measures the vigor, dedication and absorption to studying. The answers on this instrument are provided on a six-point Likert scale and range from 1—completely disagree to 6—completely agree [49]. Cronbach’s alpha for the study engagement scale in our study was α = 0.400. The instrument used for the assessment of physical activity was the International Physical Activity Questionnaire Short Form (IPAQ-SF), which examines the time spent in vigorous and moderate physical activity and time spent walking. Based on the time spent in each of the intensities of physical activity (vigorous and moderate physical activity and time spent walking), IPAQ-SF enables the calculations of energy expenditure in MET-minutes/week [50,51].

Perceived social support was examined using the multidimensional scale of perceived social support (MSPSP) [52,53,54]. This self-rating instrument refers to a subjective assessment of support from friends, family and a partner and contains 12 questions with answers on a seven-point Likert scale ranging from 1—completely disagree to 7—completely agree. The score on this scale represents the average score on each item [52,53,54]. Cronbach’s alpha for MSPSP in our study was α = 0.922.

Problematic internet use was examined using the problematic internet use questionnaire [55,56]. This 18-item scale examined behavior associated with internet use in three dimensions: obsession, neglect and control disorder and has answers provided on a five-point Likert scale ranging from 1—never to 5—always/almost always. Cronbach’s alpha for the problematic internet use questionnaire in our study was α = 0.886.

State impulsivity was examined using the state impulsivity scale [57]. This is a scale that consists of 20 items, and the answers to each of the items are provided on a four-point Likert scale ranging from 0—almost never to 3—almost always. Cronbach’s alpha for the state impulsivity scale in our study was α = 0.940.

Emotional states of depression, anxiety and stress were examined using the previously used Serbian translation of the DASS-42 questionnaire (Depression, Anxiety, Distress Scale-42) [58], which is a self-report scale that examines the negative emotional states of depression, anxiety and stress in adults and in older adolescents. The scale has 42 items with answers provided on a four-point Likert scale ranging from 0—never to 3—sometimes. For each of the scales, we used the proposed cut-offs for the 95th percentile that correspond with severe or extremely severe symptoms: 21 for depression, 15 for anxiety and 26 for stress [59]. The 95th percentile was used as a cut-off in order to help distinguish the groups with the most pronounced symptoms of depression, anxiety and stress (categories of severe and extremely severe in the original scoring) [59] and to minimize the overestimation of the prevalence of depressive, anxiety and stress symptoms in this study that uses self-reported data. Cronbach’s alpha for the entire DASS-42 scale was α = 0.969; for the depression domain (DASS-D), it was α = 0.935; for anxiety (DASS-A), it was α = 0.898; and for stress (DASS-S), it was α = 0.940.

The statistical analyses were done using analytical and descriptive statistics. The differences between the groups with depression, anxiety and stress were examined using the Chi-square test for the categorical variables and using the T-test and Mann–Whitney U-test for numerical variables. Normality was examined using the Shapiro–Wilk and Kolmogorov–Smirnov tests. Model building followed a hierarchical conceptual approach, in which variables were entered in blocks based on theoretical relevance: (1) socio-demographic characteristics, (2) health-related factors, (3) lifestyle factors, and (4) psychosocial variables. Variables from each block were retained in the model based on both statistical significance and their conceptual importance. Multicollinearity was assessed using the variance inflation factor (VIF > 5) and tolerance (<0.1). The forward Wald model was applied for the final modeling in order to reduce the possibility of overfitting the data (number of parameters per outcome event of at least ten). All analyses were done using the Statistical Package for Social Sciences SPSS 22.0.

## 3. Results

The majority of students reported living in urban areas (50.8%), more than two-thirds were female (68.1%), and more than half of them reported being single (54.5%). Around one in six students reported smoking tobacco (16.3%), 66.3% reported alcohol use, and 23.8% reported binge drinking in the past month. The socio-demographic, socio-economic and lifestyle characteristics of the participants are presented in Table 1.

A total of 61 students (9.3%) had a score on the DASS-D of ≥95th percentile for the scale indicating severe or extremely severe depression; a total of 129 (19.6%) had a score on the DASS-A of ≥95th percentile for the scale indicating severe or extremely severe anxiety; and a total of 91 (13.9%) had a score of ≥95th percentile for the scale on the DASS-S scale indicative of severe or extremely severe stress.

The participants with and without a score of ≥95th percentile for the scale on the DASS-D significantly differed in the type of residence, self-rated financial status, familial relationships, self-rated health, frequency of tobacco smoking, use of electronic cigarettes, cannabis use, use of anti-anxiety medications, mobile phone addiction, time spent on social media per day in hours, social support, problematic internet use score and state impulsivity score. The characteristics of the participants with and without a score of ≥95th percentile on the DASS-D scale are presented in Table 2.

Participants with and without a score of ≥95th percentile on the DASS-A scale differed in faculty that they are enrolled in, sex, self-rated financial status, familial relationships, self-rated health, tobacco smoking, use of tobacco-heating products, cannabis use, mobile phone addiction, score on study engagement scale, social support, score on problematic internet use scale and state impulsivity score. The differences between the participants with and without a score of ≥95th percentile on the DASS-A scale are presented in Table 3.

Participants with and without a score of ≥95th percentile on the DASS-S scale significantly differed in faculty enrolled in, sex, age, self-rated financial status, familial relationships, self-rated health, cannabis use, use of anti-anxiety medications, mobile phone addiction, time spent on social media per day, scores on the study engagement scale, social support scale, problematic internet use and state impulsivity scale. The characteristics of participants with and without a score of ≥95th percentile on the DASS-S scale are presented in Table 4.

Multivariate logistic regression analysis with a score of ≥95th percentile on the DASS-D scale as an outcome variable showed the association of depression with living in rural areas (OR: 2.21, 95% CI: 1.05–4.66), average self-rated health (OR: 5.05, 95% CI: 2.35–10.83), use of anti-anxiety medications (OR: 2.94, 95% CI: 1.31–6.61), mobile phone addiction (OR: 3.25, 95% CI: 1.55–6.81), low social support (OR: 0.68, 95% CI: 0.51–0.92) and impulsivity (OR: 1.03, 95% CI: 1.01–1.05). Multivariate logistic regression analysis with a score of ≥95th percentile on the DASS-A scale as an outcome variable showed the association between anxiety and female sex (OR: 2.59, 95% CI: 1.29–5.24), average self-rated health (OR: 4.35, 95% CI: 2.34–8.09), use of tobacco-heating products (OR: 5.63, 95% CI: 1.01–31.22), use of anti-anxiety medications (OR: 3.17, 95% CI: 1.55–6.49), score on social support scale (OR: 0.60, 95% CI: 0.45–0.80), and score on state impulsivity scale (OR: 1.04, 95% CI: 1.02–1.06). Multivariate logistic regression analysis with a score of ≥95th percentile on the DASS-S scale as an outcome variable showed the association of stress with poor female self-rated financial status (OR: 8.66, 95% CI: 2.52–29.72), average self-rated health (OR: 3.98, 95% CI: 2.03–7.81), use of anti-anxiety medications (OR: 3.50, 95% CI: 1.64–7.48), time spent on social media per day (OR: 1.18, 95% CI: 1.05–1.34) and score on the state impulsivity scale (OR: 1.04, 95% CI: 1.02–1.06). The Nagelkerke R square for the model for the score of ≥95th percentile on the DASS-D scale as an outcome variable was 0.328, for the model for score of ≥95th percentile on the DASS-A scale as an outcome variable was 0.312, and for the model for the score of ≥95th percentile on the DASS-S scale as an outcome variable was 0.288. The results of the multivariate logistic regression analyses are presented in Table 5.

## 4. Discussion

Our study showed the score above the 95th percentile on the DASS-D scale indicative of severe depression was present in just below 10% of participants; the score on the DAAS-A scale above the 95th percentile, indicative of severe anxiety, was present in one in five participants, while one in six had severe stress.

### 4.1. Depressive Symptoms

The prevalence of depressive symptoms in our study was on average two times higher compared to the commonly reported prevalence in the general population [2]. However, the prevalence in our study was lower than the prevalence of scores indicative of severe and extremely severe depressive symptoms in the study conducted among the students in the United Arab Emirates that showed the combined prevalence of the two at 31.1% among female students and 19.1% among male students [60]. Although the prevalence was slightly higher among female students in our study as well (10.1% vs. 7.7%), the difference was not significant, unlike in the previous study conducted among the Serbian population in Kosovo, where female sex was associated with higher levels of depressive symptoms [44]. Multivariate logistic regression analysis with a score of ≥95th percentile on the DASS-D scale as an outcome variable showed that students who reported living in rural areas had almost 2.5 times higher likelihood of having depression. Although the studies both on adults [61] and young adults [62] have shown that the prevalence of depression is higher in urban areas, the study that included university freshmen in China has shown higher levels of depression in those from rural areas [63]. University students do present a population that differs from the general population in terms of socio-economic characteristics and dependence on student scholarships or loans for finances, along with dependence on parents; they also face unique challenges in academic settings. Students from rural areas usually face the challenges in adapting to living in large, urban university centers that markedly differ from their place of origin [63], which all explains the higher prevalence of depression in this population and the differences in the results of the studies including the university students and the results of the studies including the general population.

In this context, the findings may also be interpreted through contemporary psychotraumatology frameworks that emphasize the role of cumulative social stressors and disrupted identity integration. Ozturk’s concept of “dissoanalysis” highlights how exposure to structural stress, social instability, and mediated environments may contribute to fragmentation of psychological integration and increased vulnerability to depressive symptomatology [64,65]. Students from rural areas, especially those originating from socio-economically disadvantaged or socially isolated settings, may experience a form of chronic stress exposure that aligns with these mechanisms.

Additionally, students in the population in our study that reported living in rural areas may be a high percentage from the Serbian enclaves in Kosovo that are facing the challenges of unemployment, poverty, and access to healthcare more commonly than those living in urban areas, pronounced by the isolation of these territories [66]. These conditions may represent not only socio-economic disadvantage but also forms of collective or contextual stress that, as suggested in recent theoretical models, can contribute to the transformation of individual distress into broader patterns of social vulnerability [67]. However, further research is needed to confirm this interpretation. Average self-rated health was also associated with five times higher likelihood for a score of ≥95th percentile on the DASS-D scale, confirming the association between general health and mental health [68]. Students that reported using anti-anxiety medications in the past 12 months were almost three times more likely to have a score of ≥95th percentile on the DASS-D scale. This is in line with previously shown higher likelihood for the use of anti-anxiety medications among students with more pronounced depressive symptoms [69]. However, we cannot determine the direction of the association in our study due to its cross-sectional design, and as we did not collect data on the indications for use of anti-anxiety medications. These students may have been using these medications as a part of regular treatment but may also be misusing the anti-anxiety medications and using them without a prescription. Further studies on the patterns of use of anti-anxiety medications and types of medications used are needed for a better understanding of this issue. Students that had mobile phone addiction had more than three times higher likelihood for a score of ≥95th percentile on the DASS-D scale, in line with previously shown results [70]. The associations between depression and mobile phone addiction were previously shown to be through the excessive checking of notifications and sleep problems associated with excessive use of mobile phones, but also with the delay in melatonin production associated with the electromagnetic waves and mobile phone lights [70]. Beyond these behavioral and physiological pathways, contemporary theoretical perspectives suggest that intensive engagement with digital media may also contribute to psychological fragmentation and difficulties in maintaining coherent self-experience, particularly in vulnerable populations [64]. Additionally, another possible explanation may be due to our cross-sectional design, as the opposite association has also been previously described, and students with higher levels of depressive symptoms may use mobile phones more with the aim of distracting themselves from the negative emotions [71].

### 4.2. Anxiety Symptoms

Almost 20% of the participants in our study had a score of ≥95th percentile on the DASS-A scale, indicative of anxiety, which is within the range identified in a systematic review that included the studies on the prevalence of anxiety among medical students (range from 7.4% to 55%) [26]. A score of ≥95th percentile on the DASS-A scale, like a score of ≥95th percentile on the DASS-D scale, was associated with average self-rated health in our study, once again indicating the complex association between general health and different mental health outcomes [68]. From a public health perspective, the rising issues with the use of anti-anxiety medications among university students should be addressed through detailed research, as it seems to be an environment in which the misuse and problematic patterns of use of these medications develop more commonly [72]. On the other hand, almost three-quarters of the participants in our study with a score of ≥95th percentile on the DASS-A scale did not report the use of anti-anxiety medications, showing the necessity for a wide screening approach for university students and thorough mental health assessment and support for this population. Higher social support was negatively associated with a score of ≥95th percentile on the DASS-A scale, supporting the previous results on the protective effects of social support on mental health [73]. Higher scores on the impulsivity scale were associated with the score of ≥95th percentile on the DASS-A scale in our study. The impulsivity was previously examined among various mental health disorders such as dependencies, bulimia nervosa, and affective disorders, although it has been traditionally assumed that anxiety and impulsivity have a negative association [74]. However, research shows that there is a common co-occurrence of anxiety and impulse control disorders and also of anxiety and affective disorders [74], showing the more complex association between anxiety symptoms and impulsivity.

### 4.3. Stress Symptoms

One in six students in our study displayed symptoms of stress, similar to the prevalence among male students in the United Arab Emirates [60]. There was no significant association between sex or age and a score of ≥95th percentile on the DASS-S scale in our study, although previous studies have shown that students in the beginning of their studies perceive higher levels of stress [75]. Our study did not include the examination of gender roles, caregiving or possible discrimination that could all potentially mediate these findings, especially in the region that is still living through the consequences of an armed conflict. Students that rated their financial status as poor were more than eight times more likely to have a score of ≥95th percentile on the DASS-S scale in our study. Students with worse financial status may be prone to stresses due to facing financial difficulties in their daily lives, due to the cost of living in university centers [76]. These students may also be working along with studying in order to decrease the financial strains; however, this may negatively influence their stress levels. As with a score of ≥95th percentile on the DASS-D and DASS-A scales, a score of ≥95th percentile on the DASS-S scale was associated with self-rated health and use of anti-anxiety medications, once again highlighting the need for the detailed assessment but also a prompt intervention for the misuse of anti-anxiety medications in this population. Rather, the interventions aiming to strengthen the protective factors and improve coping mechanisms against stress should be implemented, such as the establishment of safe platforms for discussion of the fears, promotion of inclusivity and kindness, or integration of self-care with daily routines [77]. Higher impulsivity scores were associated with a score of ≥95th percentile on the DASS-S scale in our study. Unlike common mental health issues, which seem to be a consequence of impulsivity, it was previously shown that the increased impulsivity seems to be a consequence of exposure to stress and stress symptoms, which may explain findings in our study, although further studies may be necessary to examine this association more thoroughly [78].

### 4.4. Strengths and Limitations

Our study has a few possible limitations. The first is its cross-sectional design, as it does not allow us to establish the causal relationships between the variables. Second, the symptoms of mental health issues were self-reported, so the prevalence may be over- or underestimated, especially keeping in mind that the scale used, DASS-42, is only a screening instrument and should not be considered in any way an instrument for diagnostic assessment. Additionally, the study population included only students who were present in the class on the day of the survey, and the students with chronic illnesses, work obligations or any kind of absenteeism may have been excluded, leading to possible underestimation of mental health issues. As we aimed to examine multiple factors associated with mental health, there was a possible risk of type 1 error, which we tried to mitigate with the limited number of factors included in the multiple regressions. The sample size was estimated using Slovin’s formula, which provides only an approximate calculation and does not account for specific effect sizes or model-based requirements. Therefore, the adequacy of the sample size for detecting smaller associations may be limited. Due to sample size constraints and the need to maintain model parsimony, interaction terms were not included in the final models; therefore, potential effect modification between key variables could not be assessed. However, the strengths of this study are that, to the best of our knowledge, this is the first study that examined the mental health of the university students at the University of Pristina-Kosovska Mitrovica, who present a vulnerable population due to the constant political tension in the area.

## 5. Conclusions

This study has shown a significant burden of psychological distress among students at the University of Pristina-Kosovska Mitrovica, as one in ten students reported symptoms indicative of severe depression, one in five of anxiety, and one in six of stress. The results of this study indicate the multifactorial nature of mental health. It is important to understand that this study was conducted among students of a Serbian university in the region with longstanding political tensions, which may be associated with the mental health of the population, and this should be taken into consideration when interpreting the results. Future studies should use a longitudinal design in order to establish the causal relationships between the variables and examine the possible interventions that could aim to strengthen the social support and resilience of students in this environment. The results of our study and future longitudinal studies may then help establish the need for on-campus counseling, screenings for mobile phone and internet overuse, or development and implementation of educational programs for students on the safe use of anti-anxiety medications. Further, there is a need for improved accessibility of mental health services, especially for rural areas or enclaves.

## Figures and Tables

**Table 1:** Total sample characteristics.

Characteristics	Total N (%)
Faculty (N = 657)	
Faculty of physical education	32 (4.9)
Natural and mathematical sciences faculty	47 (7.2)
Faculty of technical sciences	39 (5.9)
Teachers’ faculty	49 (7.5)
Faculty of agriculture	52 (7.9)
Faculty of economics	100 (15.2)
Faculty of medicine	188 (28.6)
Faculty of law	41 (6.2)
Faculty of philosophy	109 (16.6)
Type of residence (N = 596)	
Urban	303 (50.8)
Rural	293 (49.2)
Sex (N = 653)	
Male	208 (31.9)
Female	445 (68.1)
Age in years X ± SD (N = 615)	21.70 ± 3.97
Relationship status (N = 649)	
Single	354 (54.5)
Married/in a relationship	295 (45.5)
GPA X ± SD (N = 392)	7.94 ± 1.12
Self-rated financial status (N = 647)	
Poor	23 (3.6)
Average	253 (39.1)
Good	371 (57.3)
Family relationships (N = 648)	
Poor	14 (2.2)
Average	73 (11.3)
Good	561 (86.6)
Self-rated health (N = 633)	
Poor	13 (2.1)
Average	100 (15.8)
Good	520 (82.1)
Tobacco smoking (N = 657)	
Yes	107 (16.3)
No	550 (83.7)
Use of electronic cigarettes (N = 647)	
Yes	41 (6.3)
No	606 (93.7)
Tobacco-heating products (N = 646)	
Yes	13 (2.0)
No	633 (98.0)
Alcohol consumption (N = 646)	
Yes	428 (66.3)
No	218 (33.7)
Binge drinking in the past month (N = 588)	
Yes	140 (23.8)
No	448 (76.2)
Cannabis use (N = 634)	
Yes	28 (4.4)
No	606 (95.6)
Use of anti-anxiety medications (N = 657)	
Yes	98 (14.9)
No	559 (85.1)
Mobile phone addiction (N = 657)	
Yes	131 (19.9)
No	526 (80.1)
Time spent on social media per day X ± SD (N = 641)	4.36 ± 2.92
Time spent playing video games X ± SD (N = 488)	1.17 ± 3.06
BMI ± SD (N = 631)	22.87 ± 3.75
Study engagement X ± SD (N = 599)	35.78 ± 15.49
MET-minutes/week (N = 494)	3065.70 ± 2800.14
Social support X ± SD (N = 604)	6.06 ± 1.02
PIU score X ± SD (N = 599)	40.01 ± 12.95
Impulsivity score X ± SD (N = 570)	16.94 ± 13.62

GPA—grade point average; BMI—body mass index; PIU—problematic internet use; MET—metabolic equivalent of task.

**Table 2:** Characteristics of the participants with and without the ≥95th percentile of depression score (unadjusted analysis, Chi-square for categorical variables, the T-test and Mann–Whitney U-test for numerical variables).

Characteristics	Score of ≥95th Percentile DASS-D N (%)	Score of <95th Percentile on DASS-D Scale N (%)	*p*-Value
Faculty			0.185
Faculty of physical education	2 (6.3)	30 (93.7)	
Natural and mathematical sciences faculty	4 (8.5)	43 (91.5)	
Faculty of technical sciences	3 (7.7)	36 (92.3)	
Teachers’ faculty	4 (8.2)	45 (91.8)	
Faculty of agriculture	3 (5.8)	49 (94.2)	
Faculty of economics	3 (3.0)	97 (97.0)	
Faculty of medicine	23 (12.2)	165 (87.8)	
Faculty of law	7 (17.1)	34 (82.9)	
Faculty of philosophy	12 (11.0)	97 (89.0)	
Type of residence			0.041 *
Urban	23 (7.6)	280 (92.4)	
Rural	37 (12.6)	256 (87.4)	
Sex			0.322
Male	16 (7.7)	192 (92.3)	
Female	45 (10.1)	400 (89.9)	
Age in years X ± SD	21.17 ± 2.29	21.56 ± 3.27	0.954
Relationship status			0.960
Single	32 (9.0)	322 (91.0)	
Married/in a relationship	27 (9.2)	268 (90.8)	
GPA X ± SD	8.14 ± 1.08	7.99 ± 1.06	0.414
Self-rated financial status			0.007
Poor	5 (21.7)	18 (78.3)	
Average	30 (11.9)	223 (88.1)	
Good	24 (6.5)	347 (93.5)	
Family relationships			0.001
Poor	7 (50.0)	7 (50.0)	
Average	13 (17.8)	60 (82.2)	
Good	39 (7.0)	522 (93.0)	
Self-rated health			0.001 *
Poor	4 (30.8)	9 (69.2)	
Average	27 (27.0)	73 (73.0)	
Good	28 (5.4)	492 (94.6)	
Tobacco smoking			0.010
Yes	17 (15.9)	90 (84.1)	
No	44 (8.0)	506 (92.0)	
Use of electronic cigarettes			0.017
Yes	8 (19.5)	33 (80.5)	
No	51 (8.4)	555 (91.6)	
Tobacco-heating products			0.078
Yes	3 (23.1)	10 (76.1)	
No	56 (8.8)	577 (91.2)	
Alcohol consumption			0.259
Yes	43 (10.0)	385 (90.0)	
No	16 (7.3)	202 (92.7)	
Binge drinking in the past month			0.975
Yes	13 (9.3)	127 (90.7)	
No	42 (9.4)	406 (90.6)	
Cannabis use			0.001
Yes	8 (28.6)	20 (71.4)	
No	49 (8.1)	557 (91.9)	
Use of anti-anxiety medications			0.001 *
Yes	22 (22.4)	76 (77.6)	
No	39 (7.0)	520 (93.0)	
Mobile phone addiction			0.001 *
Yes	30 (22.9)	101 (77.1)	
No	31 (5.9)	495 (94.1)	
Time spent on social media per day X ± SD	4.50 ± 1.83	3.95 ± 2.21	0.007
Time spent playing video games X ± SD	0.83 ± 1.03	1.30 ± 4.13	0.940
BMI ± SD	23.03 ± 3.42	22.74 ± 3.70	0.309
Study engagement X ± SD	36.50 ± 7.94	34.31 ± 11.26	0.143
MET-minutes/week	2331.25 ± 2174.85	2680.68 ± 2257.72	0.201
Social support X ± SD	5.53 ± 1.09	6.13 ± 0.82	0.001
PIU score X ± SD	44.75 ± 7.66	40.99 ± 13.56	0.001
Impulsivity score X ± SD	24.33 ± 6.62	16.54 ± 12.26	0.001

MET—metabolic equivalent of task. * difference remains significant in the multivariate analyses.

**Table 3:** Characteristics of the participants with and without the ≥95th percentile of anxiety score (unadjusted analysis, Chi-square for categorical variables, the T-test and Mann–Whitney U-test for numerical variables).

Characteristics	≥95th Percentile on DASS-A N (%)	<95th Percentile on DASS-AN (%)	*p*-Value
Faculty			0.004
Faculty of physical education	3 (9.4)	29 (90.6)	
Natural and mathematical sciences faculty	10 (21.3)	37 (78.7)	
Faculty of technical sciences	6 (15.4)	33 (84.6)	
Teachers’ faculty	16 (32.7)	33 (67.3)	
Faculty of agriculture	8 (15.4)	44 (84.6)	
Faculty of economics	7 (7.0)	93 (93.0)	
Faculty of medicine	43 (22.9)	145 (77.1)	
Faculty of law	12 (29.3)	29 (70.7)	
Faculty of philosophy	24 (22.0)	85 (78.0)	
Type of residence			0.083
Urban	53 (17.5)	250 (82.5)	
Rural	68 (23.2)	225 (76.6)	
Sex			0.006
Male	28 (13.5)	180 (86.5)	
Female	101 (22.7)	344 (77.3)	
Age in years X ± SD	20.93 ± 1.90	21.68 ± 3.43	0.100
Relationship status			0.340
Single	65 (18.4)	289 (81.6)	
Married/In a relationship	63 (21.4)	232 (78.6)	
GPA X ± SD	8.14 ± 0.96	7.98 ± 1.08	0.328
Self-rated financial status			0.002
Poor	11 (47.8)	12 (52.2)	
Average	45 (17.8)	208 (82.2)	
Good	72 (19.4)	299 (80.6)	
Family relationships			0.001
Poor	8 (57.1)	6 (42.9)	
Average	22 (30.1)	51 (69.9)	
Good	98 (17.5)	463 (82.5)	
Self-rated health			0.001 *
Poor	5 (38.5)	8 (61.5)	
Average	48 (48.0)	52 (52.0)	
Good	71 (13.7)	449 (86.3)	
Tobacco smoking			0.003
Yes	32 (29.9)	75 (70.1)	
No	97 (17.6)	453 (82.4)	
Use of electronic cigarettes			0.108
Yes	12 (29.3)	29 (70.7)	
No	115 (19.0)	491 (81.0)	
Tobacco-heating products			0.015
Yes	6 (46.2)	7 (53.8)	
No	121 (19.1)	512 (80.9)	
Alcohol consumption			0.750
Yes	85 (19.9)	343 (80.1)	
No	41 (18.8)	177 (81.2)	
Binge drinking in the past month			0.377
Yes	31 (22.1)	109 (77.9)	
No	84 (18.8)	364 (81.3)	
Cannabis use			0.007
Yes	11 (39.3)	17 (60.7)	
No	113 (18.6)	493 (81.4)	
Use of anti-anxiety medications			0.001 *
Yes	37 (37.8)	61 (62.2)	
No	92 (16.5)	467 (83.5)	
Mobile phone addiction			0.001
Yes	47 (35.9)	84 (64.1)	
No	82 (15.6)	444 (84.4)	
Time spent on social media per day X ± SD	4.12 ± 2.15	3.96 ± 2.15	0.011
Time spent playing video games X ± SD	0.72 ± 1.17	1.39 ± 4.38	0.643
BMI ± SD	22.84 ± 3.50	22.75 ± 3.72	0.890
Study engagement X ± SD	32.31 ± 9.84	34.94 ± 11.29	0.019
MET-minutes/week	2197.20 ± 1756.45	2759.55 ± 2336.55	0.149
Social support X ± SD	5.74 ± 1.10	6.16 ± 0.78	0.001 *
PIU score X ± SD	44.06 ± 14.22	40.62 ± 13.00	0.001
Impulsivity score X ± SD	23.25 ± 10.30	15.70 ± 12.09	0.001 *

MET—metabolic equivalent of task. * difference remains significant in the multivariate analyses.

**Table 4:** Characteristics of the participants with and without the ≥95th percentile of stress score (unadjusted analysis, Chi-square for categorical variables, the T-test and Mann–Whitney U-test for numerical variables).

Characteristics	Score of ≥95th Percentile on DASS-S Scale N (%)	Score of <95th Percentile on DASS-S Scale N (%)	*p*-Value
Faculty			0.003
Faculty of physical education	2 (6.3)	30 (93.8)	
Natural and mathematical sciences faculty	3 (6.4)	44 (93.6)	
Faculty of technical sciences	8 (20.5)	31 (79.5)	
Teachers’ faculty	4 (8.2)	45 (91.8)	
Faculty of agriculture	4 (7.7)	48 (92.3)	
Faculty of economics	5 (5.0)	95 (95.0)	
Faculty of medicine	35 (18.6)	153 (81.4)	
Faculty of law	8 (19.5)	33 (80.5)	
Faculty of philosophy	22 (20.2)	87 (79.8)	
Type of residence			0.454
Urban	41 (13.5)	262 (86.5)	
Rural	46 (15.7)	247 (84.3)	
Sex			0.001 *
Male	14 (6.7)	194 (93.3)	
Female	77 (17.3)	368 (82.7)	
Age in years X ± SD	20.64 ± 1.68	21.70 ± 3.38	0.008 *
Relationship status			0.165
Single	43 (12.1)	311 (87.9)	
Married/In a relationship	47 (15.9)	248 (84.1)	
GPA X ± SD	8.13 ± 0.83	7.99 ± 1.10	0.623
Self-rated financial status			0.001 *
Poor	10 (43.5)	13 (56.5)	
Average	35 (13.8)	218 (86.2)	
Good	45 (12.1)	326 (87.9)	
Family relationships			0.001
Poor	4 (28.6)	10 (71.4)	
Average	19 (26.0)	54 (74.0)	
Good	67 (11.9)	494 (88.1)	
Self-rated health			0.001 *
Poor	5 (38.5)	8 (61.5)	
Average	34 (34.0)	66 (66.0)	
Good	49 (9.4)	471 (90.6)	
Tobacco smoking			0.201
Yes	19 (17.8)	88 (82.2)	
No	72 (13.1)	478 (86.9)	
Use of electronic cigarettes			0.269
Yes	8 (19.0)	34 (81.0)	
No	86 (13.2)	565 (86.8)	
Tobacco-heating products			0.865
Yes	2 (15.4)	11 (84.6)	
No	87 (13.7)	546 (86.3)	
Alcohol consumption			0.803
Yes	60 (14.0)	368 (86.0)	
No	29 (13.3)	189 (86.7)	
Binge drinking in the past month			0.628
Yes	22 (15.7)	118 (84.3)	
No	63 (14.1)	385 (85.9)	
Cannabis use			0.001
Yes	10 (35.7)	18 (64.3)	
No	77 (12.7)	529 (87.3)	
Use of anti-anxiety medications			0.001 *
Yes	29 (29.6)	69 (70.4)	
No	62 (11.1)	497 (88.9)	
Mobile phone addiction			0.001
Yes	37 (28.2)	94 (71.8)	
No	54 (10.3)	472 (89.7)	
Time spent on social media per day X ± SD	4.40 ± 2.52	3.91 ± 2.13	0.019
Time spent playing video games X ± SD	1.96 ± 5.93	1.15 ± 3.59	0.595
BMI ± SD	23.04 ± 3.23	22.72 ± 3.75	0.418
Study engagement X ± SD	34.28 ± 16.08	34.49 ± 10.06	0.024
MET-minutes/week	2582.00 ± 2006.49	2669.17 ± 2291.46	0.177
Social support X ± SD	5.84 ± 1.00	6.13 ± 0.83	0.033
PIU score X ± SD	45.40 ± 14.21	40.56 ± 13.01	0.001
Impulsivity score X ± SD	26.04 ± 10.30	15.59 ± 11.77	0.001 *

MET—metabolic equivalent of task. * difference remains significant in the multivariate analyses.

**Table 5:** Multivariate logistic regression analyses using the forward Wald method.

Characteristics	Score of ≥95th Percentile on DASS-D Scale OR (95% CI)	Score of ≥95th Percentile on DASS-A Scale OR (95% CI)	Score of ≥95th Percentile on DASS-S Scale OR (95% CI)
Type of residence			
Urban	1.0 ref.cat	/	/
Rural	2.21 (1.05–4.66)	/	/
Sex			
Male	/	1.0 ref. cat	/
Female	/	2.59 (1.29–5.24)	/
Age in years X ± SD	/	/	/
Self-rated financial status			
Poor	/	/	8.66 (2.52–29.72)
Average	/	/	1.55 (0.81–2.99)
Good	/	/	1.0 ref. cat
	/		
	/		
	/		
Self-rated health			
Poor	4.21 (0.69–25.62)	0.35 (0.04–3.26)	3.25 (0.57–18.53)
Average	5.05 (2.35–10.83)	4.35 (2.34–8.09)	3.98 (2.03–7.81)
Good	1.0 ref. cat	1.0 ref. cat	1.0 ref. cat
Tobacco-heating products			
Yes	/	5.63 (1.01–31.22)	/
No	/	1.0 ref. cat	/
	/		
	/		
Use of anti-anxiety medications			
Yes	2.94 (1.31–6.61)	3.17 (1.55–6.49)	3.50 (1.64–7.48)
No	1.0 ref. cat	1.0 ref. cat	1.0 ref. cat
Mobile phone addiction			
Yes	3.25 (1.55–6.81)	/	/
No	1.0 ref. cat	/	/
Time spent on social media per day	/	/	1.18 (1.05–1.34)
/			
Social support	0.68 (0.51–0.92)	0.60 (0.45–0.80)	/
Impulsivity score	1.03 (1.01–1.05)	1.04 (1.02–1.06)	1.04 (1.02–1.06)
Nagelkerke R square	0.328	0.312	0.288

MET—metabolic equivalent of task. ‘/’ indicates variable not entered in this model.

## Data Availability

The data can be made available upon a request to the corresponding author.
